# Core-labelling technique (CLT): a novel combination of the ingrowth-core method and tracer technique for deep root study

**DOI:** 10.1186/s13007-020-00622-4

**Published:** 2020-06-10

**Authors:** Eusun Han, Dorte Bodin Dresbøll, Kristian Thorup-Kristensen

**Affiliations:** Department of Plant and Environmental Sciences, University of Copenhagen, Højbakkegård Allé 13, 2630 Taastrup, Denmark

**Keywords:** Deep roots, Ingrowth-core, Tracer techniques, ^15^N, nutrient analogues, Alfalfa

## Abstract

**Background:**

Ingrowth-core method is a useful tool to determine fine root growth of standing crops by inserting root-free soil in mesh-bags for certain period of time. However, the root density observed by the method does not directly explain the nutrient uptake potential of crop plants as it varies over soil depth and incubation time. We have inserted an access-tube up to 4.2 m of soil depth with openings directly under crop plants, through which ingrowth-cores containing labelled soil with nutrient tracers were installed, called core-labelling technique (CLT). The main advantage of CLT would be its capacity to determine both root density and root activity from the same crop plants in deep soil layers. We tested the validity of the new method using a model crop species, alfalfa (*Medicago sativa)* against three depth-levels (1.0, 2.5 and 4.2 m), three sampling spots with varying distance (0–0.36, 0.36–0.72 and > 5 m from core-labelled spot), two sampling times (week 4 and 8), and two plant parts (young and old leaves) under two field experiments (spring and autumn).

**Results:**

Using CLT, we were able to observe both deep root growth and root activity up to 4.2 m of soil depth. Tracer concentrations revealed that there was no sign of tracer-leakage to adjacent areas which is considered to be advantageous over the generic tracer-injection. Root activity increased with longer incubation period and tracer concentrations were higher in younger leaves only for anionic tracers.

**Conclusions:**

Our results indicate that CLT can lead to a comprehensive deep root study aiming at measuring both deep root growth and root activity from the same plants. Once produced and installed, the access-tubes and ingrowth-cores can be used for a long-term period, which reduces the workload and cost for the research. Therefore, CLT has a wide range of potential applications to the research involving roots in deep soil layers, which requires further confirmation by future experiments.

## Background

Despite their potential to exploit deep plant nutrient resources [35], the dynamics of nutrient uptake by deep roots is poorly understood. In fact, the majority of root investigations carried out in arable fields are limited to the topsoil (i.e. ≤ 0.3 m) [22], while even annual crops can often grow roots to 2 m depth or deeper [15, 46].

In agro-forestry studies root development and uptake beyond 2 m of soil depth have been confirmed [8, 33], while only few agronomic studies reached beyond 2 m of soil depth (e.g. [27]). One of the common reasons for the reluctance to study deep roots is the lack of suitable methods. Root methods that can be applied for root studies beyond 2 m of soil depth are rare and expensive to adopt. Therefore, a technical development of root methods at depth is required.

Root methods can be broadly categorized into destructive and non-destructive methods. The former includes the profile wall method [3] that has been used for determination of root distribution patterns in arable subsoil [16]. Sampling procedures such as soil core (e.g. [28, 51, 57]) and monolith sampling (e.g. [36]) have been also used for determination of rooting density, rooting depth and root architecture at various spatial scales. Such destructive methods are laborious, and number of observations is often limited.

Non-destructive approaches such as the minirhizotron technique is useful for dynamic observation of deep roots, and has been widely used in agronomic field studies in relation to nutrient dynamics of crop plants [45, 53]. Recently, non-invasive technique utilizing a wide range of electromagnetic radiation for root phenotyping has been proposed [56], which however, requires further investigation in field conditions.

Ingrowth-core methods are also suitable for rapid, and thereby frequent investigation of gross growth of roots [50]. The basic concept of the technique is to place a known volume of *root*-*free* soil in a mesh-bag to which, the fine roots of standing crops grow within the known time and depths. Advantages of ingrowth-core methods can be that it reduces the uncertainty of the time interval, i.e. observers can assume that the root growth occurred during the known period of time [9]. A downside of the approach is that it can be biased as roots are growing into an initially root-free environment, which would not occur in undisturbed soil. In addition, inserting the mesh bags might disturb the root growth. However, this was proven not to be significant by investigating on the root growth near to the disturbed area by the profile wall method [50].

Tracer techniques are useful tools for studying *root activity*—as defined “the ability of the plant root to induce changes in soil close to the root” [14], which affects plants’ nutrient acquisition. This approach focuses on the plants’ capacity to acquire the given nutrients by comparing the accumulated tracer contents between labelled and un-labelled plants. ^15^N, as a stable isotope, is a well-known tracer for the study of the plant-soil interface [13], and has been used for root studies also under arable field conditions [30]. By the use of the stable isotope, N uptake has often been related to rooting density [55] and rooting depth [1]. Isotopes of K and S also exist, but due to high costs, nutrient analogues are often used. Among nutrient analogues Li has been recognized for its usefulness as an analogue to K. One of the pioneering studies by Martin et al. [37] concluded on the validity of Li as a tracer after experiments in a barley and field bean intercropping set-up. Cs [59] and Rb [7] as monovalent cations are also known to have the same uptake mechanism as K. As an analogue to S, Se is used as it is taken up via high affinity sulphate transporters and have similar uptake pathways as S in plant roots [52]. In addition, radioactive tracers such as ^33^P and ^32^P were adopted for P uptake studies [12, 41], especially with mycorrhizae [42], which however, requires safer handling of the radioactivity in field conditions.

Applying tracers by injection include a risk of tracer mobility, especially in case of high rainfall after labelling [17]. This requires extra efforts to reduce the leaching risk, e.g. covering the plot surface, or to verify the movement of tracers by analyzing soil samples adjacent to the injected spots. Therefore, a secure way of labelling with a minimized risk of leaching or escaping of tracers will be helpful in tracer studies.

Incubation time of ingrowth-cores has been suggested in a wide range of periods; less than 4 weeks under actively growing annual crops (e.g. barley and potato) due to the mortality of fine roots [50], and longer than 6–9 months in boreal and temperate coniferous forests [26, 54]. However, no field experiments have been conducted to test the optimum incubation time for determining the root growth and shoot-labelling status, and none in arable subsoils.

In tracer studies the rooting density is often determined on adjacent plants and not the plants receiving the tracer [8]. This separated approach for root uptake vs. root density investigation can potentially lead to a misleading interpretation of the data. Distribution patterns of soil nutrients and roots are highly heterogeneous, especially, in the subsoil [22, 60], and as a result the spots for tracer application and shoot biomass collection might not have the same conditions as the spots for root measurements.

Therefore, a feasible method that can allow us to measure the root characteristics and nutrient accumulation by the shoot of the same plants in situ is called for. The aim of this study was to develop a method for measuring root growth and activity within the same soil volume beyond 2 m of soil depth. For this purpose, we have combined the ingrowth-core method with tracer-labelling and developed the core-labelling technique (CLT). Alfalfa (*Medicago sativa* L.) was chosen as a model plant to test the CLT, as it is known for its deep-rooting capacity.

The new approach should meet the following criteria:

- To quantify deep root growth beyond 2 m of soil depth;
- To allow precise location and time of tracer application;
- To allow root extraction of the labelled soil volume after the end of the experiment.

Therefore, we have constructed long sloping metal access-tubes with openings that are placed directly under plants, through which ingrowth-cores with labelled-soil are installed. We hypothesize that (i) the alfalfa roots will grow into the ingrowth-cores within a 60 days period and that tracer concentration will increase in the shoot due to the uptake; (ii) the CLT with its containment can precisely locate the labelled spot, so only the targe-plants growing directly above the labelled-spots are labelled; (iii) tracer concentration is higher after 8 weeks than 4 weeks due to the prolonged root growth period and thereby increased accessibility to the labelled soil; (iv) the CLT can demonstrate relationships between root density and root uptake activity.

## Materials and methods

### Experimental site

**Fig. 1:**
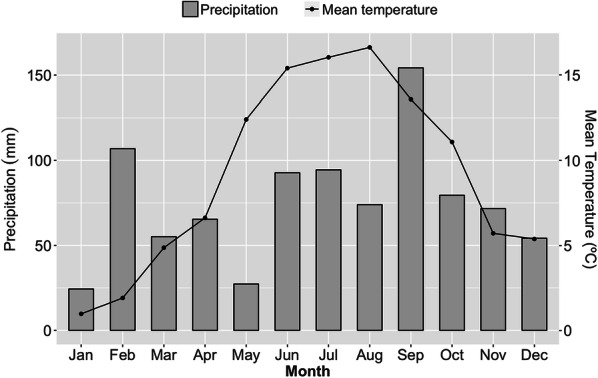
Precipitation (mm) and mean temperature in 2017 at the study site

A field trial was established at the experimental station of the University of Copenhagen in Taastrup, Denmark (55° 40′ N; 12° 18′ E). The soil was an Agrudalf soil classified as sandy loam according to the ISSS classification. Detailed description on the soil physical and chemical condition at the study site is available in Table 1. A soil profile observation was done by national soil survey of Denmark in Tune, approximately 8 km away from the study site consisting of the same glacial till material. According to the survey, the soil profile consists of six horizons, viz., Ap (0–25 cm), Bt (25–43 cm), Bt (g) (43–66 cm), Cc(g)1 (66–95 cm), Cc(g) (95–116 cm) and Ceg (> 116 cm). Weather data were collected at a weather station 0.3 km from the experimental site (Fig. 1). The temperature and precipitation were measured at 2 and 1.5 meters-height, respectively.

**Table 1:** Physical and chemical soil characteristics at the study site

Soil type	Soil depth (m)	pH	Clay (%)	Silt (%)	Fine sand (%)	Coarse sand (%)	Bulk density (g cm^−3^)	P (%)	K (%)
Ingrowth-core soil		8.1	12.5	12.4	45.5	28.9		0.004	0.041
Field soil	0–0.25	7.6	13.0	15.2	42.3	27.8	1.56	0.042	0.110
	0.25–0.75	7.8	20.2	12.9	40.3	26.1	1.64	0.013	0.084
	0.75–1.5	4.5	19.9	16.3	37.8	25.9	1.76	0.006	0.065
	1.5–3.0	8.2	19.3	18.9	36.6	25.1	1.77	< 0.004	0.074
	3.0–4.5	8.1	19.0	25.9	33.1	21.7	1.77	0.004	0.111

### Design and installation of access-tubes and ingrowth-cores

**Fig. 2:**
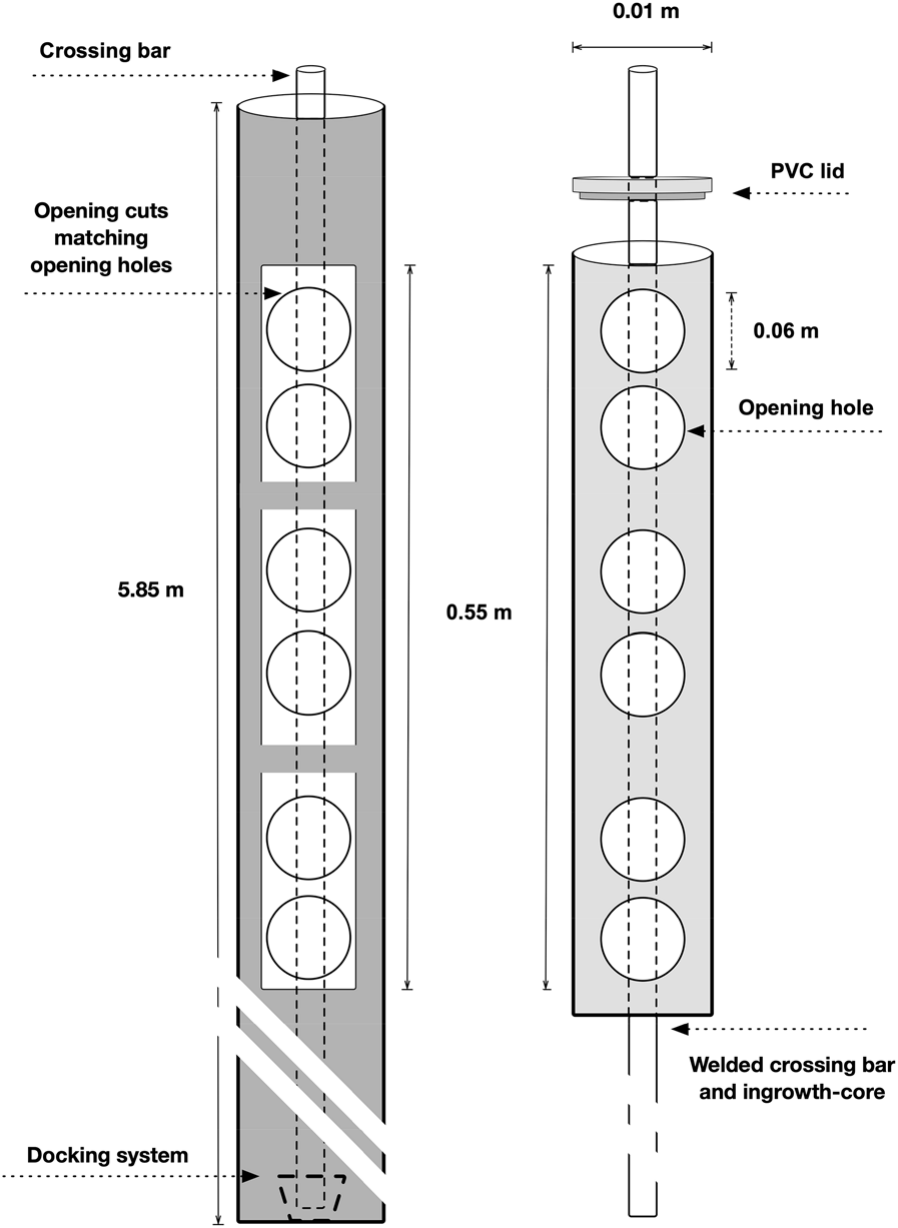
Schematic diagrams of an access-tube with ingrowth-core inserted (left) and an ingrowth-core (right)

**Fig. 3:**
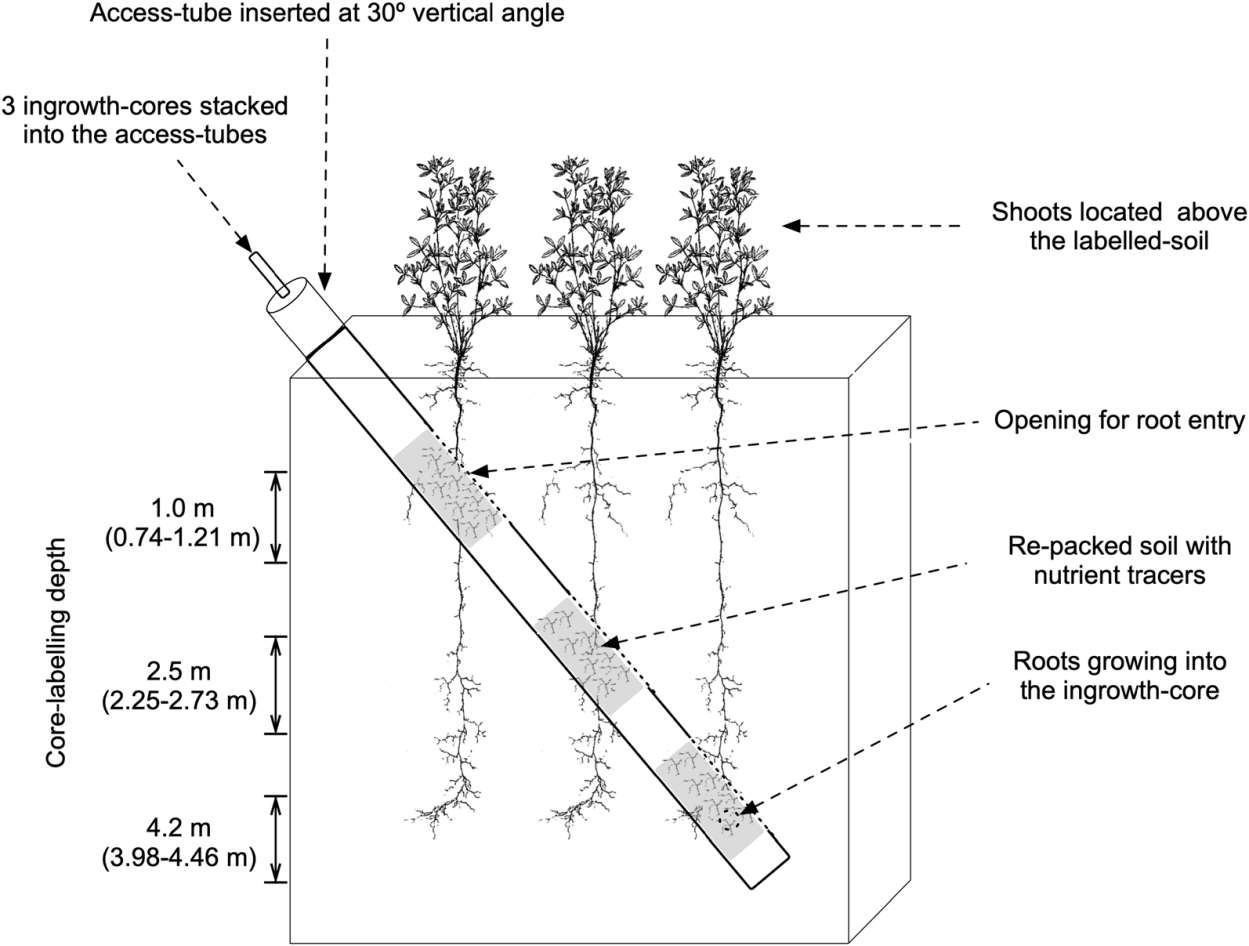
A schematic diagram describing the concept of core-labelling technique (CLT); Figures of plants are from Kutschera et al. [31]

A stainless-steel access-tube (Fig. 2; left) with a total length of 5.85 m and an inner-diameter of 0.01 m was inserted using a spiral auger (Arkill Holding, Denmark) into the soil at 30° from the vertical line (Fig. 3). It had 0.55 m-long openings at three intervals at 0.85–1.40 m, 2.6–3.15 m and 4.6–5.15 m. Due to the 30° angle, it created three corresponding soil depth-levels, vertically; 1.0 m (0.74–1.21 m), 2.5 m (2.25–2.73 m) and 4.2 m (3.98–4.46 m). The ingrowth-cores were developed as stainless-steel tube structures with openings, designed to be filled and re-packed with tracer-labelled soil and inserted into the access-tubes (Fig. 2; right). One ingrowth-core had a container of 0.55 m-long with an inner-diameter of 0.01 m. In the center of each ingrowth-core structure a thinner steel tube was placed, resulting in a net ingrowth-core volume of 3931 cm^3^. Each ingrowth-cores had 6 circular openings with a diameter of 0.06 m (Fig. 2; right). The openings of ingrowth-cores were designed to match those of access-tubes (Fig. 2; right) through which, the plant roots from the bulk soil can access the soil inside the ingrowth-cores. A steel rod system was developed for the insertion and retraction of three ingrowth-cores into each access-tube. The crossing bar inside the ingrowth-core was needed to provide stability upon stacking the ingrowth-cores and also to regulate the distance between them (see Fig. 2). At the bottom of the access-tube, a cylinder-shaped docking-system was installed as a guiding system for the placement of the ingrowth-cores into the access-tubes (Fig. 2; left). The top end of the two lower cores had a corresponding structure, so that the ingrowth-cores could be locked on top of each other. The reason for having the pre-installed semi-permanent access-tubes was to promote simpler and less laborious insertion/extraction of ingrowth-cores for long-term field trials, which otherwise require soil-drilling each time.

### Experimental design

Alfalfa (*M. sativa* L. ‘Creno’) was sown on Sep 9, 2015. The seeding density was 2.5 g m^−2^. The crop was mowed three times per season. No fertilizer was applied during its growth. Two experiments were performed in 2017; one in spring (4 May–4 July) and the other in autumn (31 Aug–2 Nov). Access-tubes were inserted at the plot heads of six alfalfa plots (1.5 m × 10 m) leading to six replicates. In the spring experiment, due to a soil collapse in one access-tube, the replicates were reduced to five.

### Experimental procedure

#### Core-labelling

^15^N, Li, Cs, Rb and Se were used as nutrient tracers. They were prepared from ^15^NH_4_Cl, Li_2_CO_3_, Cs_2_CO_3_, Na_2_SeO_4_ and Rb_2_CO_3_ with the amount of 275.24 mg, 210.78 mg, 728.26 mg, 0.474 mg, 535.02 mg per ingrowth-core, respectively. Except for ^15^N, the assumed enrichment levels were derived from Hoekstra et al. [17]. The tracers were prepared in solution form and mixed with a subsoil medium. While re-packing the labelled soil in the spring, a high soil strength was created to avoid soil spillage with a bulk density of 1.78 g cm^−3^. At the second experiment in autumn, we applied a glass-fiber mesh covering the holes in the ingrowth-cores and lowered the bulk density to 1.44 g cm^−3^. The soil used for the ingrowth-cores was a sandy loam subsoil taken from below 0.5 m (Roskilde Stone & Gravel Ltd.). Physical and chemical characteristics of subsoil medium are available in Table 1. After re-packing is done, the ingrowth-cores were inserted into the access-tubes.

#### Shoot sampling and measurement

Two shoot samplings took place in both experiments at week 4 and 8 after the core-labelling. At week 4 young and old leaves were collected, separately, whereas the entire biomass was collected as a whole at week 8.

**Fig. 4:**
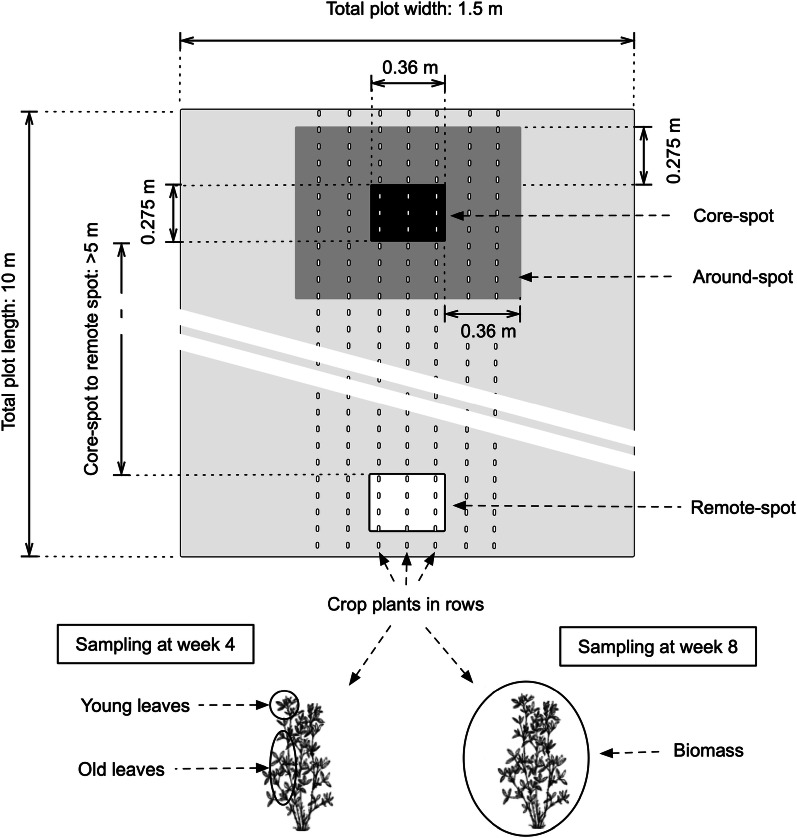
A schematic diagram on shoot sampling strategies. In spring experiment: shoot samples for core-spot (black-shaded area) were collected within 0.275 m × 0.360 m directly above the ingrowth-cores. Samples from around-spot (grey-shaded area) were collected around the core-spot (0.361–0.720 m). Shoot samples from remote-spot (white-shaded area) were collected > 5 m away from the core-spots. In both experiments: shoots were sampled twice (week 4 and week 8). At week 4, young and old leaves were collected separately, whereas the entire biomass was collected at week 8

Samples were collected directly above each of the three ingrowth-cores. Due to the insertion angle, each 0.55 m ingrowth-core had the corresponding length on the surface of 0.275 m (Fig. 4). Although the diameter of the ingrowth-core was 0.1 m, we targeted at the width that corresponded to three crop rows (equivalent to 0.36 m). Thus, the total area of sampling per opening was 0.275 m × 0.360 m on the soil surface for both experiments. In the spring experiment, shoot samples were collected from three different spots. Firstly, shoots were sampled as described at the targeted areas directly above the labelled ingrowth-cores (core-spot; 0–0.36 m). In addition, the area around the core-spot was sampled (around-spot; 0.361–0.720 m), and as a control, shoot samples at least 5 m away from the core-spot were collected (remote-spot).

The collected samples were oven-dried at 85 °C for 48 h, and ground for further analysis. Stable isotopic ratios of N (^15^N) was measured at Stable Isotope Facility, UC Davis, using a ThermoScientific GasBench-Precon gas concentration system interfaced to a Thermo Scientific™ DELTA V™ Plus isotope-ratio mass spectrometer (Bremen, Germany). Upon analysis of the nutrient analogues, the samples were microwave-digested in nitric acid (70%). For the spring experiment, the sample digests were analyzed by Inductively Coupled Plasma Mass Spectrometry (ICP-MS, Thermo Scientific iCAP-Q equipped with CCTED; collision cell technology with energy discrimination, Bremen, Germany). In the autumn experiment, the digests were analyzed using ICP Sector Field Mass Spectrometry (ICP-SFMS, ELEMENT XR, Thermo Scientific, Bremen, Germany) using a combination of internal standardization and external calibration.

In the autumn experiment, the tracer concentrations from the core-spots were subtracted to the tracer concentrations from the remote-spot to calculate the excess tracer concentrations (dTC).

#### Root sampling and measurement

The ingrowth-cores were retracted for root sampling after 8 weeks of core-labelling and the soil samples were stored in a cooling room (5 °C) until the extraction. The entire volume of the soil re-packed into ingrowth-cores was taken out and visible roots were separated by root washing. The clean root samples were scanned on a flatbed scanner (Epson Perfection V700). The resulting root images (600 dots per inch; DPI) were analyzed with the ‘WinRHIZO Pro’ (Version 2016c, 32 Bit) software. Minimum surface area of the object was set for 2 cm^2^, and minimum length to width ratio of the root objects to 2. Medium image smoothening was chosen for noise removal. Root-length density (RLD; cm cm^−3^), root diameter (mm) were obtained from the images. Root biomass (RBM; mg cm^−3^) was determined after drying the root samples for 48 h at 85 °C in the oven. Specific root length (SRL; m g^−1^) was calculated based on RBM and RLD.

### Statistical analysis

R version 3.4.1 R Core [44] was used for statistical analysis. The package lme4 [2] was used for linear mixed-effects model analysis [43]. For the root traits (RLD, RBM, root diameter, and SRL) the effects of core-labelling depth—1.0 and 2.5 m for the spring experiment and 1.0, 2.5 and 4.2 m for the autumn experiment, were tested. In the spring experiment, the effects of sampling distance (core-, around- and remote-spot) and core-labelling depth (1.0 and 2.5 m) on the tracer concentrations in aboveground biomass were tested. In autumn experiment, the effects of sampling time (week 4 and 8) and core-labelling depth (1.0, 2.5 and 4.2 m) on excess tracer concentrations (dTC) were tested. The effects of sampling part (young and old leaves) on the tracer concentrations measured after 4 weeks of core-labelling were tested in the spring and autumn experiments.

Main effects and interactions were tested for significance (P≤ 0.05) based on the approximated degrees of freedom calculated by the package lmerTest [32]. Differences were considered significant at P < 0.05. Tukey test P-values for pairwise comparisons were adjusted for multiplicity, by single step correction to control the family-wise error rate, using the multcomp package [19].

## Results

### Installation, cost and labour requirement for implementation of CLT

Depending on the soil and weather conditions, the soil-drilling using a spiral auger and the insertion of access-tubes allowed the installation of 6-8 access-tubes per day with two labours. It indicates that one day was sufficient to install the required number of access-tubes for this study, which resulted in a cost of 7000 Euros (52,080 Danish Krone). Six days by one professional were required to produce 18 ingrowth-cores for the given study with a cost of 2830 Euros (21,120 Danish Krone).

Labelling of soil with the nutrient tracers and re-packing of the soil into 18 ingrowth-cores required less than a day with three labours. Insertion of the ingrowth-cores into the access-tubes required three labours, which was done in a day. We were able to finish the shoot sampling in a day with two labours. Extraction of ingrowth-cores required three labours and an electronic winch mounted on a tractor, and it required one day to complete.

### Alfalfa root growth into ingrowth-cores

**Fig. 5:**
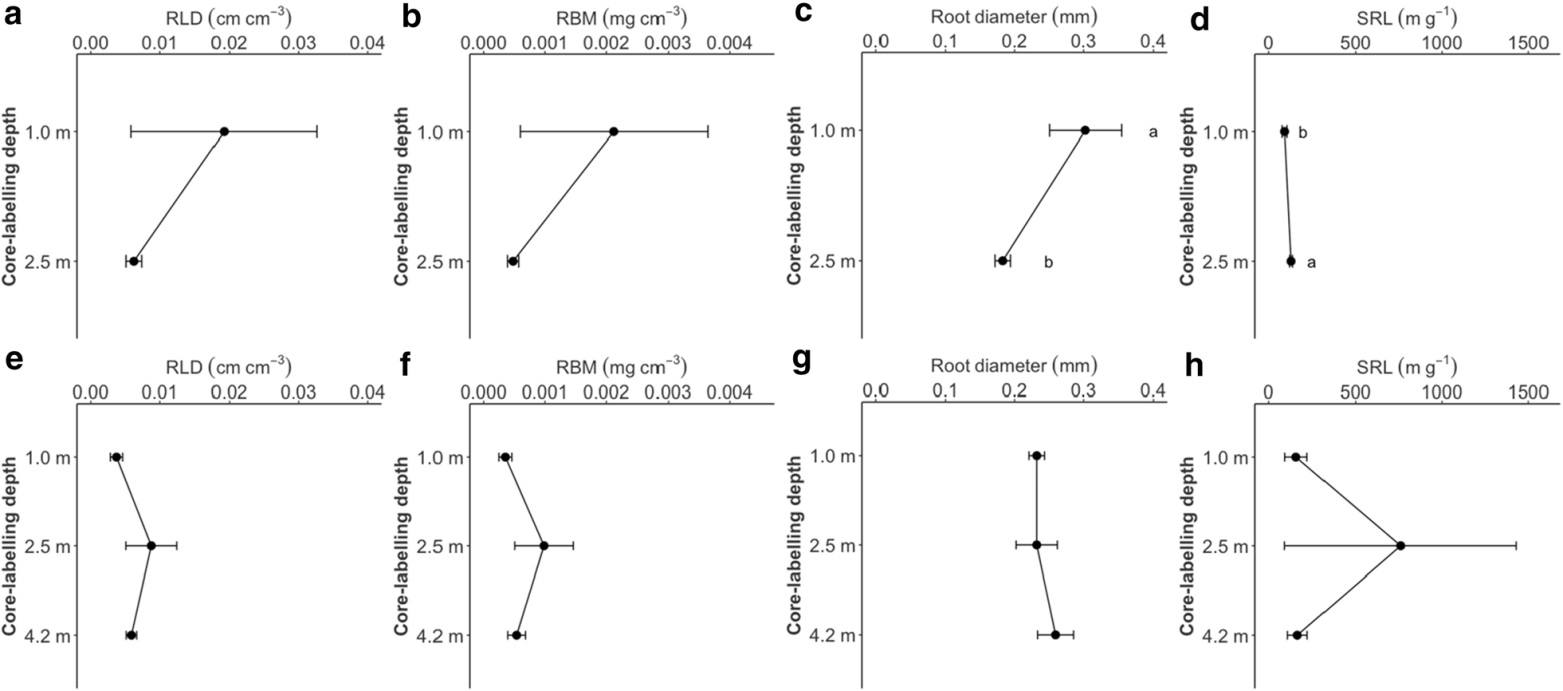
Root-length density (RLD; cm cm^−3^), root biomass (RBM; mg cm^−3^), root diameter (mm) and specific root-length (SRL; g m^−1^) of alfalfa affected by core-labelling depth measured in the spring (**a**–**d**) and autumn experiment (**e**–**h**). Small letters indicate significant differences between the treatments (Tukey HSD; *P *≤ 0.05). Means and one SE are shown

Roots of alfalfa grew into ingrowth-cores at all depths. The density-based traits such as RLD and RBM exhibited low amount and did not significantly differ between soil depths in either of the seasons (Fig. 5a, b, e and f). The variability in RLD in the spring experiment was high, from 0.003 to 0.082 cm cm^−3^ at 1.0 m depth. In spring, the root diameter decreased significantly with depth, which led to the significant effect of depth on SRL. No differences in root diameter and SLR between the depth was found in the autumn (Fig. 5g and h).

### Effect of sampling spot on tracer concentrations

**Fig. 6:**
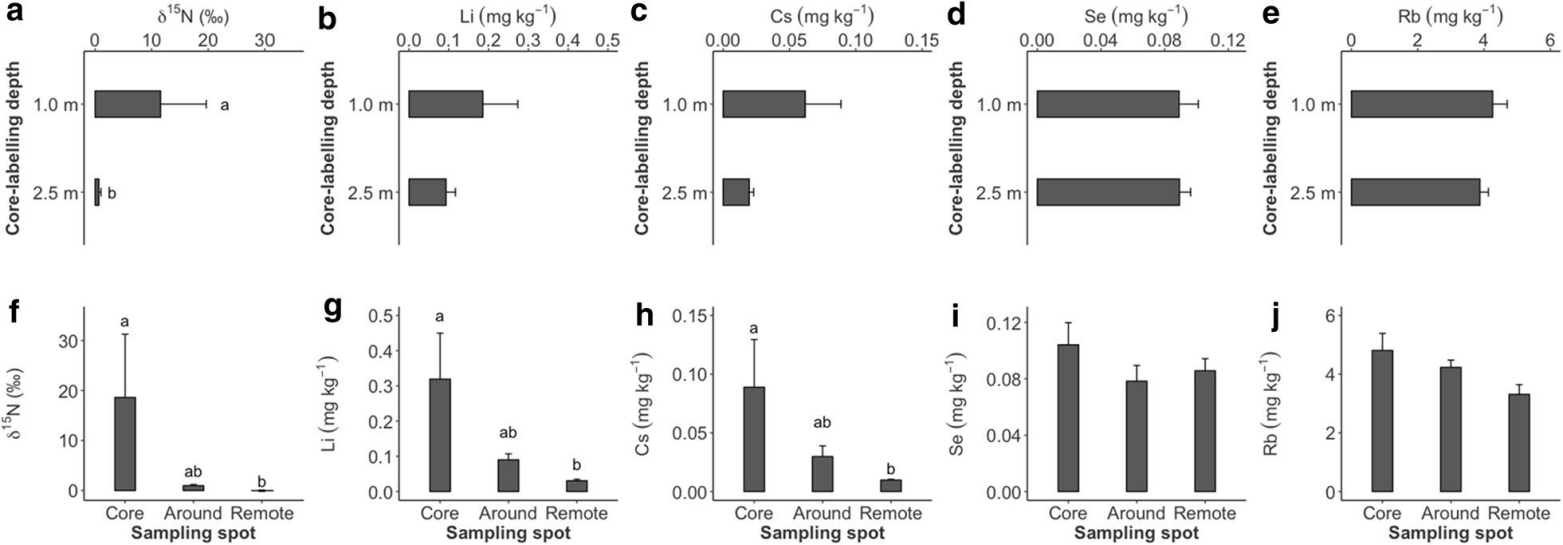
Tracer concentration of ^15^N, Li, Cs, Se and Rb in alfalfa shoot biomass affected by core-labelling depth (1.0 and 2.5 m; **a**–**e**) sampling distance (core-, around- and remote-spot; **f**–**j**) in the spring experiment. Roman letters indicate significant differences between the treatments (HSD Tukey; *P *≤ 0.05). Means and one SE are shown

Concentration of tracers at different sampling spot with varying distance (core-spot, around-spot and remote-spot) and core-labelling depth (1.0 m and 2.5 m) is presented here based on the data from the spring experiment. According to mixed-effects model analysis, core-labelling depth significantly affected tracer concentrations of ^15^N (Fig. 6a) while Li, Cs, Se and Rb were unaffected (Fig. 6b–e). Tracer concentrations of ^15^N, Cs and Li were significantly affected by sampling spot. Se and Rb concentrations were affected by neither factor (Fig. 6d, e, i and j).

Multiple comparisons on tracer concentrations between the sampling spots were carried out. Tracer concentrations of ^15^N, Li and Cs (Fig. 6f–h) were significantly higher at the core-spot compared to the remote-spot across the core-labelling depths, meanwhile the around-spot exhibited moderate differences. Se and Rb concentrations revealed decreases from the crop-spot to remote-spot, however, the differences were not significant (Fig. 6i and j).

### Effect of sampling time on excess tracer concentrations (dTC)

**Fig. 7:**
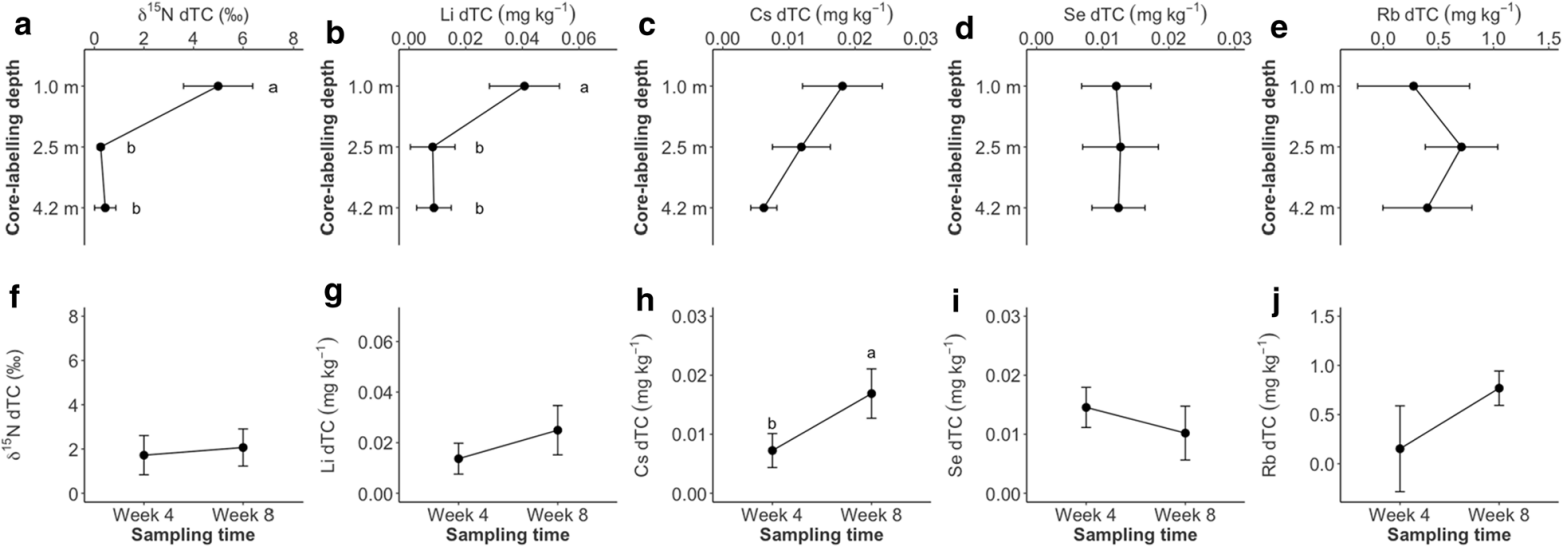
Excess tracer concentrations (dTC) of ^15^N, Li, Cs, Se and Rb from alfalfa shoot biomass affected by core-labelling depth (1.0, 2.5, 4.2 m; **a**–**e**) and sampling time (Week 4 and 8; **f**–**j**) in the autumn experiment. Roman letters indicate significant differences between the treatments (HSD Tukey; *P *≤ 0.05). Means and one SE are shown

In the autumn experiment, excess tracer concentrations (dTC) at three core-labelling depths (1.0, 2.5 and 4.2 m) at two sampling times (week 4 and week 8) were analyzed. Regardless of core-labelling depth the dTC of ^15^N and Li was significantly affected by the core-labelling depth, in which, 1.0 m depth revealed significantly higher dTC (Fig. 7a and b). The difference between 2.5 and 4.2 m was insignificant. Cs dTC also revealed similar decrease along the depth, but the difference was not significant (Fig. 7c). Se dTC and Rb dTC were not affected by core-labelling depth (Fig. 7c, d and e). Cs dTC was significantly higher at week 8 compared with week 4 (Fig. 7h). Although ^15^N, Li and Rb (Fig. 7f, g and j) showed similar increase in dTC over the sampling time, the difference was not significant. Rb revealed a mild decrease in dTC over the sampling time, but the effects were insignificant.

### Effects of sampling part on tracer concentration

**Fig. 8:**
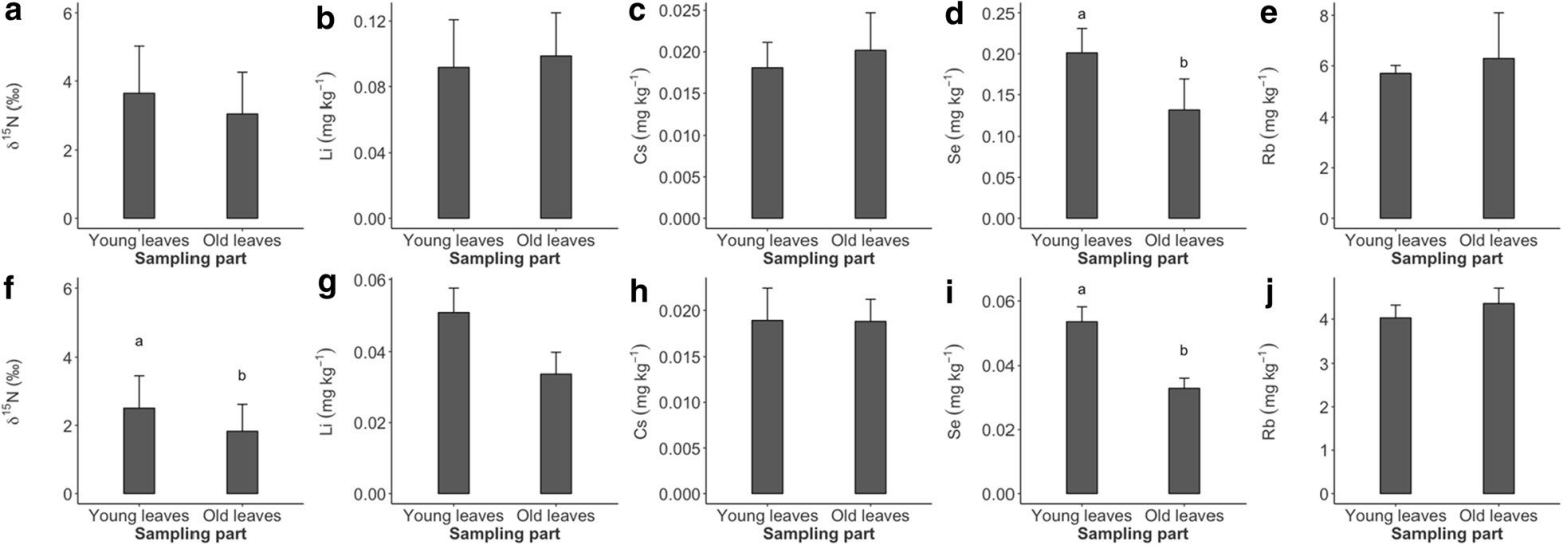
Tracer concentration of ^15^N, Li, Cs, Se and Rb in alfalfa shoot biomass affected by sampling part (young and old leaves) collected 4 weeks after the core-labelling in spring (**a**–**e**) and autumn experiment (**f**–**j**). Roman letters indicate significant differences between the sampling part (HSD Tukey; *P *≤ 0.05), respectively. Statistical analysis was done with log-transformed variables. Means and one SE are shown

In both experiments, shoot samples were separately collected from young and old leaves to compare the tracer concentrations between the sampling parts. As shown in Fig. 8, the anionic tracers, i.e., ^15^N and Se, revealed significant differences in tracer concentrations between the sampling parts—Se in both experiments (Fig. 8d and i) and ^15^N in spring experiment (Fig. 8f). No other tracers exhibited the effects of sampling part (see Fig. 8b, c, e, g, h and j).

### Root density vs. tracer concentration

**Fig. 9:**
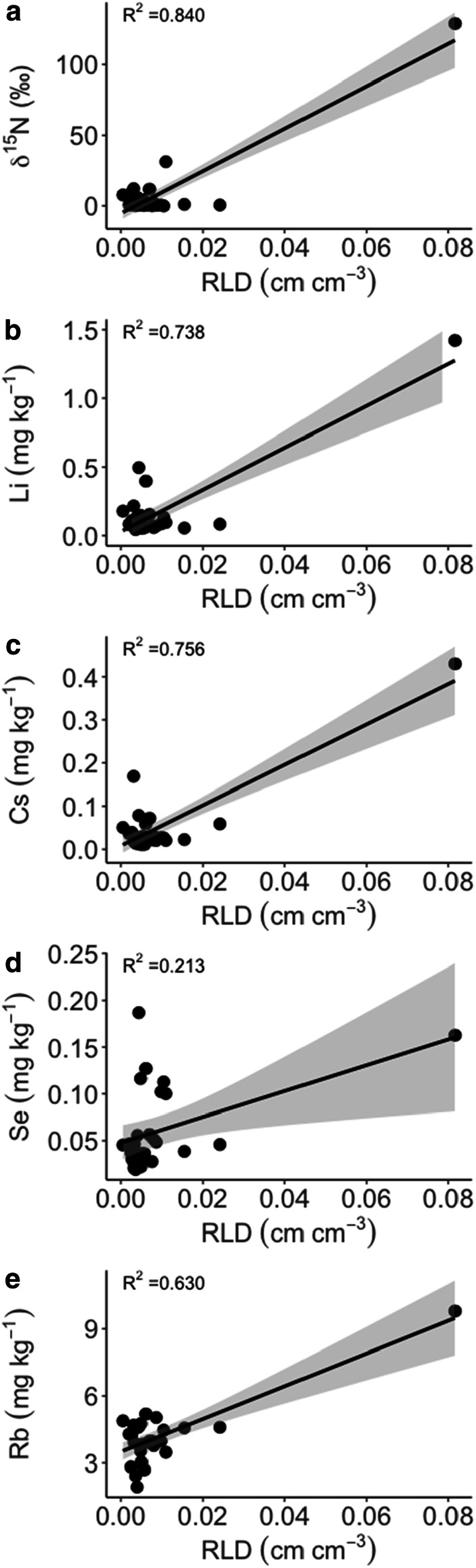
Linear regression between root-length density (RLD; cm cm^−3^) and nutrient tracers (^15^N, Li, Cs, Se and Rb). The shaded area represents one SE

We have found low values for both tracer concentrations and root density. Nevertheless, the relationships between RLD and tracers were significant (*P* ≤ 0.05). R^2^ values between RLDs and ^15^N, Li, Cs, Se and Rb were 0.840, 0.738, 0.756, 0.213 and 0.630, respectively (Fig. 9a–e). However, only few data points with high x and y values have driven the linear relationship.

## Discussion

Using CLT, root activity was detected in the aboveground biomass above the core-labelled spot and the effects of core-labelling were also moderately shown up to 0.72 m of distance horizontally. Maximum root activity was shown earlier for the mobile anionic tracers compared to the less mobile cationic tracers. The relationship between rooting density and tracer uptake was positively correlated but considering the data distribution, it was rather inconclusive.

### Deep root growth into the ingrowth-cores

As hypothesized (i), CLT, with its deep access-tubes and ingrowth-core installation, was capable of capturing deep root growth below 2 m depth. To our knowledge, this study illustrates the deepest application of an ingrowth-core technique, which, has commonly been used to a maximum of 0.5 m soil depth [5, 50]. We found alfalfa root growth and activity at 4.2 m depth in autumn, which exceeds the deepest observation on alfalfa roots, so far, done by Weaver [58] at 3.7 m after 6-years cultivation of the species. In a Haplic Luvisol, perennial crops, such as alfalfa, chicory and tall fescue were found to have deep roots when observed to 2 m of soil depth [15, 24]. When grown as mixture with *Festuca pratensis* and *Phleum pratense*, alfalfa resulted in 1.5 m of maximum rooting depth [48]. In extreme cases soil water depletion has shown root activity of alfalfa deep roots up to 10 m of soil depth [10, 34].

The deep-rooting of alfalfa in our study site must be attributed to its taproot system with a high penetration capacity [38]. The species is also known to create soil biopores [15]. The increase in biopore density formed by the penetration of large-sized taproots [23, 39] might be responsible for establishment of deep roots. Another reason for deep rooting might be the less frequent mowing done at the study site (max. 3 cuts per season) which might have enhanced the rapid root growth [58]. Finally, the deep roots found could also be a result of methodological issues. Inserting tubes into the soil always poses the risk of disturbance e.g. leaving gaps between the tube and the soil facilitating root growth along the tube.

In contrast to the high rooting depth, we have found lower rooting density of alfalfa than expected. According to our observation, the ground water level at the study site was above 2.5 m depth during the incubation period, which might have affected the increase in rooting density. The frequency of sampling, in general, is perceived as 2–4 weeks of time to ensure minimum root mortality of the ingrown fine roots [6, 50]. In our study, we doubled the time, to allow time for tracer uptake after roots had grown into the ingrowth-cores. Therefore, it is hard to conclude if the sampled roots after 60 days were at the peak in terms of density. Moreover, as indicated in Table 1, we have used same soil batch for re-packing regardless of the depth-levels for ingrowth-core installation. As a result, there were differences in soil texture between bulk soil and the re-packed soil, especially at depth (> 3.0 m), which might have retarded root growth into the ingrowth-cores due to the discontinuity in soil properties [4].

The surprising result that there were no significant differences in RLD between the three depths is expected to be a result of the high data variability, which might have been caused by heterogeneous root distribution in the deep soil layers [22] and the aforementioned limitation exerted by installation disturbance.

### Horizontal and vertical variation in root activity

Our hypothesis (ii) was only partially met. We have identified the labelled area using the CLT as intended, but the range of labelling area was larger than we assumed. We have found a tendency that tracer concentration to be elevated at the around-spot. Considering the containment-capacity of CLT the uptake of tracers at around-spot should be interpreted as acquisition by the extended root system of the neighbor plants. This is also in agreement with previous studies. For example, when Li was applied as a tracer into 50 cm of soil depth at 327 ppm (2 g of LiCl in 1000 g of soil), the adjacent maize plants up to 1 m distance were able to access the tracer [47]. However, it is uncertain to which extent the horizontal root proliferation happens when the deep roots are exposed to different conditions, such as soil compaction. One of the disadvantages of the generic injection techniques is its susceptibility for horizontal and vertical movement of the tracer solution after the injection, especially, due to high rainfall. In Hoekstra et al. [17], it was found that high rainfall (78 mm at the study site) during the incubation period caused increased concentration of the tracers (Cs, Rb and Sr) below the injection depth. Based on our observation in the spring, such leakage of tracers from the ingrowth-cores did not happen or happened to a minimum extent.

In the spring experiment, root activity did not differ significantly between the soil depths. Nevertheless, root activity tended to be higher at the upper soil layers as the rooting density in upper layers tended to be higher, especially in spring. Despite the different depth-scale applied compared to the current study, Hoekstra et al. [17] found that the depth-wise difference in plant excess tracer concentration (dTC) and recovery rates of Cs between 0.05 and 0.2 m was more than 5-folds when tested on a grass mixture. This is close to our findings in the spring experiment. The tracer concentration fell by a factor of 4.5 when the ingrowth-core depth was increased from 1.0 to 2.5 m in our experiment. Similarly, using Sr, Rb and Li, injection of tracer solutions at 0.05 m showed significantly higher root activity of seven grassland-species compared with injections at 0.15 and 0.25 m [11].

### Temporal variation in root activity

The significant increases in excess tracer concentration (dTC) of Cs over the sampling time (from week 4 to 8) partially confirms our hypothesis (iii) stating that tracer concentration after longer incubation time is higher compared to shorter time. However, the time of sampling did not alter ^15^N and Se dTC. Considering the concentration differences between the young and old leaves, it is clear that the anionic tracers, i.e., ^15^N and Se, seem to be taken up by deep roots within 4 weeks of incubation, whereas the cationic tracers required longer period time.

In autumn experiment, regardless of sampling time, dTC of ^15^N, Li, Cs and Se revealed positive values indicating the tracer was taken up from week 4 or even earlier. However, tracer accumulation was not concentrated in younger leaves at that time for Li and Cs. This might be attributed to the accumulation pathways of the cations. Especially, Li is known to be firstly accumulated in the roots, then translocated to the old leaves and does not escape the location owing to the incapacity for phloem transport [25]. Overall, it can be suggested that the optimum period of incubation for ^15^N and Se is within 4 weeks, while the other tracers need longer time for optimal uptake and measurement. Therefore, we suggest that the optimum period for incubation in CLT should differ based on the tracers used.

Based on the data on the positive dTC values in autumn experiment (except for Rb at 1.0 m), we assume that the tracer uptake happened up to 4.2 m of soil depth. However, we are also cautious to conclude on the uptake at depths, especially for Se and Rb. Firstly, no distinctive differences in dTC Se and dTC Rb between the soil depth may imply that the magnitude of tracer uptake was at very minimum level. Moreover, although we observed no indication of sharing ingrowth-cores between the plants at different depth-levels, at ground level the plants were separated by less than 1 m horizontally, which might have allowed a sharing of ingrowth-cores between plant from different depth-treatments, i.e. plants above ingrowth-cores inserted at 4.2 m were able to reach the ingrowth-cored inserted at 2.5 m. Moreover, the overall concentrations of Se and Rb at the labelled area was low. Therefore, it is possible that even the small amount of Se and Rb snatched from upper-soil layers (e.g. from 2.5 m-labelled ingrowth-core) might have been substantial to indicate the significant effects of labelling.

### Root density vs. tracer uptake

Our hypothesis (iv) on root density vs. tracer uptake was not confirmed by our CLT results. Despite the statistical indication by the linear regression, the relationship between root density and tracer uptake does not seem to be straight forward. The observed relationship was driven by one observation combining high root density with high uptake of all five tracers. First of all, we aimed to detect root activity at depth-levels where a low root growth was expected and shown. In addition, we did not substantially increase the enrichment levels of the nutrient tracers compared to other studies done on highly active root zones [18]. The reason was to avoid to affect root growth and activity of the target plants due to high concentrations of the applied chemicals [29], e.g. Toxicity in plants [49]. As a result, we had to work with a low labelling level close to the background levels and thereby a low increase in tracer concentration, which might have affected the relationship between the root density and shoot tracer concentration.

Hoekstra et al. [17] found that increasing injection density from 36 to 144 injections m^−2^ can reduce variability of tracer concentrations. It is difficult to compare this to the injection density of our approach. Yet considering the sampling area created per ingrowth-core (0.099 m^2^), it is approximately 10 injection points per m^−2^, which is substantially lower than previous studies (e.g. > 190 injections per m^2^ [21, 40]), hence the risk for high variation in tracer concentration. One question remaining is, if the detected rooting density inside the ingrowth-cores would have been enough to draw the large proportion of tracer applied. In general, RLD higher than 0.1 cm cm^−3^ is a pre-requisite for a sufficient soil water extraction [20], which might be why the higher RLD values drove the linear relationship strongly in our study.

### Potential and improvement of the CLT method

Our study shows the potential of the core-labelling technique (CLT) for studying deep nutrient uptake under field conditions, using either nutrient tracers (^15^N) or nutrient analogue tracers. Once produced and installed, the access-tubes and ingrowth-cores can be used for a long-term period, which can substantially reduce the workload and cost for research afterwards.

Despite the promising results, we have also identified some drawbacks of the approach. First of all, we have observed that the soil at the opening areas of access-tubes occasionally collapsed and left a dent, which potentially reduced bulk soil to re-packed soil contact. This might explain the large variation in root growth. Therefore, the design of the openings in the access-tubes may be adjusted to decrease the risk of soil-collapse while still allowing roots to grow across from the bulk soil to the ingrowth-core soil.

Moreover, due to the steep insertion angle the access-tube openings only left a short distance between the core-spots on the soil surface. This means that we cannot exclude the possibility of horizontal root growth and uptake from the shallower layers of plants supposedly taking tracers up from the deeper soil layers. However, as roots were found in all ingrowth-cores we expect the uptake to originate from there. The possible effect of horizontal growth could in future studies be determined by adding different tracers at the different depths and not using it for multiple tracer studies. Alternatively, a less steep insertion angle would separate the aboveground core-spots more but would require much longer access-tubes leading to increased potential of disturbance and impracticality upon insertion and extraction of the ingrowth-cores.

Except for ^15^N, one question which remain is the effect of background concentration of the elements used as tracers on the results. In general, it is recommended to have information on their level of availability in the soil, before deciding the amount of label to apply to the ingrowth-core soil.

## Conclusions

Our results suggest that CLT can be used to detect root activity of deep roots in arable fields. The approach combines deep-drilled permanent access-tubes installed at an angle, and portable ingrowth-cores used for placing labelled soil at different soil depths. Using CLT, researchers can locate the labelled spots underneath and be used to study spatial and temporal variation in root activity. The method has good potential for answering both basic and applied research questions about roots and their activity in deep soil layers. Further studies involving more realistic root-soil interface for determining deep root activity are required confirm the contribution deep roots to crop nutrient dynamics.

## Data Availability

The data will be stored on an online self-repository.
